# Digital learning in nursing education: lessons from the COVID-19 lockdown

**DOI:** 10.1186/s12912-024-02312-1

**Published:** 2024-09-11

**Authors:** Gro Gade Haanes, Etty Nilsen, Randi Mofossbakke, Marianne Wighus, Monika Ravik

**Affiliations:** 1 Faculty of Health and Social Sciences, Institute for Nursing and Health Science, University of Southeastern Norway, Campus Bakkenteigen, Horten, 3184 Borre, Norway; 2 USN Research Group of Clinical Competence, Department of Nursing and Health Sciences, Faculty of Health and Social Sciences, University of Southeastern, University of Southeastern Norway, Porsgrunn, Norway

**Keywords:** COVID-19, Digital education, E-learning, Healthcare education, Nursing education, Pandemic impact, Remote learning, Transition to e-learning

## Abstract

**Background:**

The COVID-19 pandemic necessitated a swift transition to e-learning, significantly impacting nursing education due to its reliance on practical, hands-on experiences and the critical role nurses play in healthcare. Nursing students need to achieve high levels of clinical competence through experiences traditionally obtained in clinical settings, which e-learning had to replicate or supplement. Understanding the unique challenges faced by nursing students in e-learning environments is crucial for developing educational strategies that enhance learning outcomes and contribute to improved patient care. This study aimed to explore the experiences of nursing students and newly qualified nurses (as students) with e-learning during the COVID-19 lockdown, focusing on how it influenced their learning and professional development.

**Method:**

This exploratory and descriptive study employed qualitative interviews with 31 participants, including full-time nursing students, part-time nursing students, and newly qualified nurses (as nursing students). Conducted online via Zoom during February and March 2022.

**Results:**

The findings suggest that integrating small group interactions and employing strategic pedagogical support can enhance e-learning effectiveness. However, barriers such as technological difficulties, psychological challenges, and social isolation were also identified. Understanding these unique opportunities and challenges can help educational institutions optimize e-learning strategies, ensuring nursing students are well-prepared for their crucial roles in healthcare.

**Conclusion:**

The rapid shift to e-learning due to the COVID-19 pandemic presented challenges such as technological, psychological and social aspects, but also opportunities to rethink and enhance nursing education delivery. Implementing appropriate pedagogical e-learning strategies, such as scaffolding and small group learning, can better prepare nursing students for their essential roles in healthcare. This study contributes to the body of knowledge on digital education and provides a foundation for future research aimed at optimizing e-learning in nursing education.

**Supplementary Information:**

The online version contains supplementary material available at 10.1186/s12912-024-02312-1.

## Background

The COVID-19 pandemic necessitated a rapid and unprecedented transition to e-learning across various educational disciplines, impacting fields that rely heavily on practical training, such as nursing. The abrupt shift to digital learning modalities highlighted the critical need for nursing students to achieve high levels of clinical competence through experiences traditionally obtained in clinical settings, which now had to be replicated or supplemented with e-learning [2, 3].

E-learning, broadly defined as the use of electronic media and devices to facilitate learning, emerged as a crucial tool during the pandemic, enabling the continuation of education while minimizing virus transmission risks [4]. The literature reveals varied student experiences with e-learning, emphasizing benefits such as flexibility and accessibility, yet also highlighting challenges, particularly in maintaining clinical competencies and psychological well-being [5–7].

Literature on e-learning in nursing education has highlighted a variety of student experiences, emphasizing the benefits of flexibility, accessibility, and the potential for self-paced learning [8, 9]. It is, however, important to recognize that e-learning encompasses more than just flexibility. Nursing education typically combines clinical and theoretical components [10], both of which were significantly affected during the pandemic. Barret [11] found that the COVID-19 pandemic had a detrimental impact on nursing education as a whole, with nursing students facing unprecedented challenges in areas such as academic requirements, additional clinical commitments, and personal safety measures. The theoretical component had to be completed entirely through e-learning on online platforms, without in-person interactions with educators and peers [2]. Furthermore, the challenges included inadequate digital infrastructure, inadequate experience of educators with teaching using technology, and difficulties in engaging nursing students on digital platforms [12]. Additionally, the clinical component faced barriers due to physical restrictions that reduced the ability of students to engage in clinical practice [2]. Some students and educators worry that e-learning formats may not effectively replicate the hands-on clinical experiences. In the study by Ravik et al. [2], there were a concern about the adequacy of e-learning in fulfilling the practical and interpersonal skill development that is central to nursing education. Also, nurse mentors at practice locations had increased responsibilities related to the pandemic, which reduced their availability for students [11]. Studies have revealed that nursing students exhibited decreased motivation during the pandemic that reduced their ability to acquire knowledge and skills [13, 14] Some students also experienced delays in their education, resulting in extended clinical placement periods or the omission of certain training components, leading to increased stress [15]. Given the critical role that nurses play in healthcare systems, understanding the unique challenges they face in e-learning environments is important [16]. However, e-learnings efficacy in nursing education, which combines theoretical and clinical components, remains underexplored.

This study aims to explore the nuanced experiences of nursing students and newly qualified nurses (as nursing students) with e-learning during the COVID-19 lockdown, focusing on how this transition influenced their learning and professional development. While the study primarily focuses on e-learning during the pandemic, it also considers the unique challenges and opportunities presented by the sudden shift to this mode of learning.

The integration of Vygotsky’s sociocultural theory and Marton & Säljö’s learning approaches provides a theoretical framework to understand these experiences [17, 18]. Vygotsky’s concept of the zone of proximal development (ZPD) emphasizes the role of social interaction and guided learning in achieving higher cognitive functions [17]. In the context of e-learning, this underscores the importance of structured and supportive online environments. Marton & Säljö’s distinction of how nursing students engage with digital approaches further informs our understanding of how nursing students engage with digital learning materials [18].

This study addresses significant gaps in the existing literature by providing a comprehensive exploration of the longitudinal influence of e-learning on nursing students’ clinical competencies, academic performance and psychological well-being. By examining the rapid adaptation of e-learning during the pandemic, we aim to inform future educational strategies that enhance learning outcomes and contribute to improved patient care.

## Methods

### Design

This study is a part of a larger investigation exploring nursing students learning during the COVID-19 pandemic. We employed an exploratory and descriptive research design utilizing in-depth qualitative individual and pair interviews [19]. The exploratory design was chosen to investigate the nuanced experiences of nursing students and newly qualified nurses (as nursing students) transitioning to e-learning during the COVID-19 pandemic inductively and without any theoretical approaches [20]. This approach allowed for the systematic gathering of in-depth insight into an under-researched area, particularly in response to the unprecedented global health crisis. The descriptive aspect aimed to provide a comprehensive understanding of these experiences, capturing emerging themes and patterns during data collection and analysis.

### Sample and recruitment

Our participants were recruited using a purposive sampling strategy, targeting nursing students and newly qualified nurses from one Norwegian university and various clinical placements [21]. Recruitment was facilitated via email invitations and postings on relevant educational and professional online forums, with a detailed explanation of the study’s purpose and the voluntary nature of participation.

The study included 31 participants divided into 3 distinct samples: nine full-time nursing students in their 3rd year, 12 part-time students in their fourth year, and ten 10 newly qualified nurses who had completed their education during the pandemic (Table 1). The selection of these groups was guided by the principle of information power, ensuring a rich and diversified representation of experiences to reach data saturation [22].

**Table 1:** Overview of the participants

	Participants		
	Sample 1	Sample 2	Sample 3
*Number of participants interviewed*	9 full-time students in their 3rd (and last) years of education	12 part-time students in their 4th (and last) years of education	10 newly qualified nurses who had received their 2nd and 3rd years of education during the COVID-19 lockdown
*Number of interviews*	9 individual interviews	8 individual and 2 pair interviews	10 individual interviews
*Experiences* with practice *during the lockdown*	The students missed their first practice in a nursing home or home care	The students mostly had all of their practices, but were absent during periods due to lockdowns or infection outbreaks	Several of the newly graduate nurses missed most of their hospital practice during the education process (medical, surgery, or both)
*Experiences with digital learning during the lockdown*	Digital classes Theory lectures	Digital classes Theory lectures	Digital classes Theory lectures

The inclusion of newly qualified nurses who had completed their education during the pandemic provided a unique perspective on the use of digital learning throughout their education. In addition, certain parts of their practice were replaced with digital classes, which reportedly are difficult to implement as replacements for hands-on experience [2].

### Data collection

Data were collected through semi-structured interviews conducted online via Zoom during February and March 2022 due to pandemic restrictions. The interview guide, developed by the research team, focused on experiences and reflections related to e-learning and physical presence during the education process. Each interview lasted 60–90 min and was recorded and transcribed verbatim. Demographic data were collected at the beginning of each interview using a structured questionnaire, ensuring comprehensive analysis of participants’ experiences in relation to their backgrounds. The required information power was reached after conducting 29 interviews [22].

### Ensuring trustworthiness

The study adhered to the Consolidated Criteria for Reporting Qualitative Research (COREQ) checklist to ensure methodological rigor [23, 24]. This included strategies to enhance credibility, transferability, dependability, and confirmability [25]. Researches engaged in reflexivity, examining their perspectives and potential biases to ensure they did not unduly influence the data collection and analysis.

### Analysis

Data analysis was conducted using inductive qualitative content analysis as described by Graneheim and Lundman [26] and Lindgren et al. [27] to condense extensive text into easy-to-understand pieces of information and to identify essential patterns. The process involved six steps: initial reading to identify core messages, data filtering to extract relevant text segments, data condensation into meaningful units, coding, formation of subcategories, and development of overarching theme. This method allowed for both manifest and latent content to be transformed into codes, subcategories, and categories, which were then synthesized into an overarching theme [28]. We also included illustrative quotes to support our research findings, making necessary adjustments for clarity. The whole research group took part in the analysis and discussed and agreed upon both categories and subcategories as well as the overarching theme.

### Ethics approval and consent to participate

This study was approved by the Norwegian Social Data Service (project number 396247) and adhered to the ethical principles outlined by the National Committee for Research Ethics in the Social Sciences and the Humanities [1]. Informed consent was obtained from all participants, and data were securely stored on the university’s research server.

## Findings

The study revealed three main categories and six subcategories that aligned with the overarching theme identified in the study (Table 2).

**Table 2:** Overarching theme, main theme, and subcategories

Overarching theme: Learning and Knowledge Development during E-Learning	
**Main categories**	**Subcategories**
*1. Learning possibilities and learning barriers*	***Group Dynamics in E-Learning*** *Description*: This subcategory explores how the size and composition of learning groups (small vs. large) influence interaction, engagement, and knowledge acquisition
	***Engagement in Virtual Settings*** *Description*: This subcategory explores the analysis of student participation levels during digital classes, with a focus on the impact of breakout rooms and passive learning environments.
*2. Technological challenges*	**Platform Utilization and Interface Challenges** *Description*: This subcategory addresses the issues related to the use of digital platforms like Canvas, including interface usability and consistency across different courses.
	**Connectivity and Technical Reliability** *Description*: This subcategory explores the discussion of technical difficulties encountered by students and educators, such as internet disruptions and equipment malfunctions.
*3. Psychological and social challenges (impact)*	**Cognitive and Emotional Engagement** *Description*: This subcategory examines how e-learning affects students’ ability to concentrate and maintain motivation over prolonged periods
	**Social Interaction and Isolation** *Description*: This subcategory explores the evaluation of the loss of social interaction on student well-being and the dynamics of virtual socialization in an academic context

### Learning possibilities and learning barriers

In exploring the *Learning Possibilities and Learning Barriers* of e-learning, this study categorizes the findings into two pivotal subcategories: *Group Dynamics in E-Learning* and *Engagement in Virtual Settings*. Each category sheds light on different aspects of the educational experience under the conditions imposed by the pandemic.

Participants reported that they prepared for the digital classes in the same way as they would for in-person classes. However, they felt that the lack of nonverbal cues and other communication differences sometimes reduced their ability to participate fully. Despite attendance being mandatory, participants did not report any significant benefits to digital classes over traditional in-person classes. Sometimes the students had been told to read or watch something in advance, such as a PowerPoint presentation or a film, as in a “flipped classroom,” which they reportedly were keen to do more often since this also could lead to greater engagement. They specifically mentioned that a film had the advantage of being able to be stopped and rewound if they did not understand parts or even all of it.

#### Group dynamics in E-Learning

This subcategory explores how the size and composition of learning groups (small vs. large) influence interaction, engagement, and knowledge acquisition.

The participants noted that smaller groups tend to increase the sense of responsibility that individuals had to contribute to academic discussions. One of the participants said:

*“It was easier for people to speak up*,* and there was more discussion and reflection*,* so to speak.” (2–12).*

The participants recognized the importance of peer interactions in gaining new perspectives and challenging their own ideas through reflection. They reported that listening to the experiences of others could increase their understanding and moments of realization. Additionally, the respondents found it useful to discuss the tasks performed by their peers, although some expressed reluctance to share their own assignments. One of the participants said:

*“It was also instructive to discuss other students’ cases*,* but it was clear that some were hesitant to present their cases and assignments in front of everyone else. Nevertheless*,* I believe that most students were happy about it when they received constructive feedback*,* and it was instructive.” (1–8).*

#### Engagement in virtual settings

Analysis of student participation levels during digital classes, with a focus on the impact of breakout rooms and passive learning environments.

When preparing for digital classes and group sessions, students mainly focused on their assigned tasks and prepared questions for the teacher. During group sessions, students reviewed their work and contemplated how to present or discuss their findings with their peers. Nevertheless, some students considered that the discussions during these sessions were unproductive and superficial. Some of the participants were passive, lacked motivation, and did not take the initiative to lead discussions. This resulted in some of them perceiving breakout room sessions as a waste of time:

*What kept us going in these hours*,* in the digital hours*,* was that we knew we were going into these small breakout rooms. Then we got a little more sense of responsibility to follow along and participate in the teaching*,* compared with it becoming very passive*,* with only lectures without any discussion in between.” (1–6)*.

While others were looking forward to participating in these sessions. Examples of these views are as follows:

*“During breakout room sessions*,* most students appeared exhausted due to the prolonged screen time. This led to a lack of active participation*,* with only a few students engaging in the discussions while others found excuses to be passive.” (1–5)*.

Regarding the use of black screens during digital classes, the participants felt uncomfortable about allowing others into their personal space, particularly when many students were present. They also found it inappropriate to have private activities and distractions visible in the background during academic sessions. Furthermore, they observed that distractions such as children or pets were common among students who did not use black screens. While the use of black screens was a personal choice, some participants deemed this necessary to prevent privacy invasion and distractions during digital classes:

*“There was an option not to turn on the camera*,* and many chose that. It was the easiest option*,* and people felt they were still following along even without a picture. I felt very exposed when I had my camera on. So*,* in the end*,* most people chose not to have their cameras on at all. It was the easiest choice.” (1–3)*.

Some participants considered it disrespectful when they attended classes with their cameras off and did not actively engage in the discussion:

*“The teachers said they thought it was good to be able to see us*,* and I agree that it’s probably better for learning to be able to see each other. But at the same time*,* it’s uncomfortable to sit there and not know who is looking at you at that moment*,* because you don’t know…it makes you very self-conscious. It was nice to just be able to sit at home in peace*,* and enjoy a cup of tea*,* and eat at the same time.” (1–4)*.

### Technological challenges

In addressing the *Technological Challenges* faced during the transition to e-learning, our study delves into the complexities of digital *Platform utilization and Interface challenges*, and the various *Connectivity and Technical Reliability* that significantly impacted the learning process. This main category is divided into two critical subcategories that collectively explore the infrastructural and operational issues encountered by both students and educators.

The participants reported mixed experiences with the digital classes. The lack of technical proficiency among some teachers caused delays and mistakes. At the start of the pandemic, several of the clinical placements were not ready to participate in Zoom classes, which created additional stress for those who relied on their own 3G/4G data.

The participants experienced that the university overall as well as its teachers and students were not prepared for the sudden lockdown. Some of the participants experienced confusion during a hybrid lecture in which some individuals were present in the lecture room while others had to participate remotely via Zoom:

*“Suddenly*,* the technical equipment at the university didn’t work in the lecture hall*,* or if there were things that needed to be demonstrated*,* they were too small. Or things were written on the board*,* while those who were present online had a PowerPoint file*. *So*,* there were some challenges*,* but as long as you communicated with the lecturer*,* it went well.” (2–3)*.

#### Platform utilization and interface challenges

This subcategory focuses on the practical difficulties experienced by participants of digital learning platforms such as Canvas. Issues such as user interface complexity, inconsistent usage across courses, and the steep learning curves to understand their impact on the e-learning experience.

The participants complained that teachers from different subjects used the digital platform in different ways, and that it took too long working out how to use it.

When asked about their experiences of using the digital teaching and learning platform, the participants found it easy to use, but they criticized some teachers for lacking proficiency in using the platform. They found it unacceptable for teachers to make mistakes with the platform, especially when it came to submitting important assignments. The participants wanted all teachers to use Canvas in the same way and suggested having a video showing how to do this. Many participants found the Canvas layout to be messy, and some participants wondered if all teachers understood it as well as they should have. Sometimes what the participants were looking for was hidden inside other documents, and the teachers used different terms and different file names for the same things. Sometimes there was a reference to a folder that led to another folder, making it harder for the participants to find what they needed. Related information was in various places, and the participants thought that there should be fixed places where the same types of information were posted. One of the participants said:

*“It was messy and I’m a little unsure if all the teachers have actually understood it 100% themselves. Because if there is one topic*,* a teacher has chosen to put the information in one place. So*,* then you expect that for the next topic*,* the information will be in the same place*,* but suddenly…no*,* it’s not there anymore. Apparently*,* it’s in a completely different place now. To be completely honest*,* I didn’t learn how to use Canvas properly until now*,* my fourth year.” (1–2)*.

Some subjects had schedules that were divided into weeks, while others did not have a common structure, which caused confusion.

#### Connectivity and technical reliability

Connectivity and technical reliability are crucial for successful e-learning. This subcategory explores the nature of technological disruptions like internet connectivity problems, hardware failures, and software glitches that have posed significant barriers to continuous and effective learning.

Several minor technical problems occurred during teaching sessions, such as losing the Internet connection occasionally. There were also occasions when several municipalities where the students lived triggered alarms without warning, which could interrupt entire lectures. There were also times when the private networks that the students accessed where they lived were not optimal, and several of the students reported that there was no one they could ask for IT help. The university was closed, and they also did not have access to a printer when they needed one. One of the participants said:

*“There have been these small technical problems. One thing is that you might lose the Internet connection during a lecture. There have been times when the municipality suddenly checks that the alarms work*,* which sound 10 times. And then*,* if I’m on placement and need to do a middle or end evaluation online*,* there have been issues where our private computers do not have a strong enough connection due to their ‘guest passwords’*,* and when we log in to their computers*,* they do not have a camera. They may also have very poor speakers*,* so it’s hard to hear the teacher. So*,* it has been a challenge.” (2–7)*.

Other technical problems included participants using a smartphone instead of a PC and the smartphone logging them out or being disturbed by other incoming announcements from the smartphone at the same time.

Some participants could not access the Zoom sessions due to not knowing the required passwords. This resulted in several messages going back and forth to fellow students about them not being allowed into classes that they had requested access to. This of course disrupted the teaching and took the focus away from the actual taught topic:

“Suddenly, a password was required to enter the lecture or meeting without prior notice. I also don’t think the teacher was aware of it.” (1–6)

### Psychological and social challenges

In exploring the *Psychological and Social Challenges* associated with e-learning during the pandemic, this category addresses the emotional and social dimensions that affect student engagement and learning outcomes. It is divided into two subcategories that assess *Cognitive and Emotional Engagement* of shifting from traditional classroom environments to virtual learning spaces *and Social Interaction and Isolation.*

Participants noted that the teachers increasingly did not attend practice periods, and so the students had to rely on their practice supervisor, if they had one. Some of the participants also said that the quality of digital support and guidance they received varied markedly depending on how familiar individual teachers were with using online tools.

A lack of structure and guidance in teaching arrangements and the discussion groups contributed to some of the participants considering the group sessions to be ineffective, leaving them feeling disconnected and unengaged.

The participants argued that during the pandemic, nursing students had to complete a “corona task” as part of their education program to make up for missed practical hours, which involved writing 500 words per day for each day of absence from the clinical setting. The tasks were specific to the type of care the students were providing, such as palliative care. Each task was graded as a pass or fail, and the students had to adhere to the university’s formatting and content guidelines. Participants expressed concern that the extra workload—which was additional to their existing academic and clinical responsibilities—placed them at risk of failing the course if they became ill. The participants also noted that the degree of leniency when they became ill varied between teachers. Moreover, only some of the students had prior experience of academic writing. One of the participants said:

*“Not only did you become ill and were afraid of failing*,* but you also had to perform a task that you didn’t know if you could complete*,* in addition to everything else. So*,* you are right that it also puts you at risk of failing. For example*,* XX wrote his task while he was sick*,* but the teachers preferred that he recovered first and then completed it. I think that’s a good arrangement*,* but at the same time there’s also a time pressure if you don’t get it approved before the end of the clinical practice.” (2–8)*.

#### Cognitive and emotional engagement

This subcategory examines the cognitive load and emotional strain placed on students navigating e-learning. It delves into issues such as difficulty in maintaining concentration, motivation levels, and the general mental fatigue associated with prolonged digital interaction, reflecting on how these factors hinder the learning process.

The participants widely considered long lectures on Zoom to be boring and hence difficult for maintaining concentration, especially when the teacher was simply reading out from a document. Some of the participants who found it difficult to follow the e-learning process did not want to ask for help:

*“I found it challenging to maintain structure and routines using my own initiative and responsibility during the digital classes. It was quite exhausting to have to stay at home with the uncertainty surrounding the pandemic and my own academic progress.” (3–10)*.

While others consciously took a break and established a recovery regime. Two of the participants said:

*“I took 10-minute exercise breaks to energize and refresh myself*,* as sitting in front of a computer for hours caused physical discomfort and headaches. Despite these measures*,* it remained challenging to maintain focus and motivation to review the material before and after class due to the taxing nature of e-learning.” (1–9)*.

Maintaining concentration during the long times spent in front of a computer screen while attending full-day digital classes presented a significant challenge. Although breaks were provided, many participants tended to use this time to browse the Internet with their smartphones or watch television, leading to disengagement and a loss of focus during lectures. One of the participants said:

*“The online format required more effort to concentrate compared with traditional in-person classes due to the ease of accessing other distractions such as smartphones and browsing the Internet during lectures. Consequently*,* I found it necessary to repeat material outside of class to compensate for the lack of focus during the lectures.” (3–8)*.

#### Social interaction and isolation

Focusing on the loss of physical classroom dynamics, this subcategory explores the influences of reduced face-to-face interactions. It assesses how isolation and the lack of informal social exchanges impact students’ sense of community and overall mental well-being in an academic setting.

Several of the participants also said that their motivation had sometimes been lower during this period because they did not feel any social belonging or connection with the other students when they sat in their respective dormitories or homes. The students missed coming to campus and talking to other students as a large group and discussing different topics. Showing up on campus together with the other students while preparing for examinations or writing notes and other activities could have helped them. Similarly, there were some who said that they failed examinations due to their motivation sometimes being extremely low, while others also saw upsides. One of the participants said:

*“The loss of motivation was quickly apparent when transitioning to e-learning*,* as there were several distractions at home that made it difficult to stay focused.” (1–3)*.

They had not chosen to take an e-learning course themselves, but rather such an education program had been forced on them when they were supposed to attend in person. Having to deal with a course that was almost entirely online was obviously challenging, even though they could also see that there were advantages. One of the participants said:

*“I mean*,* you have a choice of taking an online course or a physical course*,* and we have chosen a physical course. So*,* it is clear that it is something…yes. A big upheaval. But still*,* I will honestly admit that personally*,* I have found it very comfortable and actually very convenient. You get a lot of opportunities to do other things between classes*,* right? You are not necessarily bound to be at school. That’s good. The only thing is that it has affected the social aspect*,* and that*,* on the other hand*,* is something that I find challenging. Not meeting fellow students and being able to discuss and*,* you know*,* be together.” (1–4)*.

In traditional courses students have the opportunity to socialize and interact with their peers and teachers before, during, and after lectures, in person. They can also ask the teacher questions. However, the shift to e-learning resulted in the atmosphere becoming serious and somewhat intimidating. It was not as easy to ask the teacher questions before and after the lecture. Although students could see and recognize their peers on Zoom, not everyone actively participated in the discussions. While some were engaged and shared their thoughts, others became invisible and passive. This was similar to being in a physical lecture hall, but in-person interactions allowed for more opportunities to connect with and form impressions of others. This was perceived as a loss:

*“I think it’s very sad that I have lost…study buddies*,* and I haven’t gotten any student environment or anything like that. So*,* studying has become very lonely*,* and with that*,* I feel that I have lost a lot of learning opportunities.” (3 − 2)*.

Some of the participants acknowledged that digital learning can be quite individualistic, and those who only worked on their own might have missed out on opportunities to learn from others.

## Discussion

The aim of this study was to explore the nuanced experiences of nursing students and newly qualified nurses (as/while nursing students) with e-learning during the COVID-19 lockdown, focusing on how this transition influenced their learning and professional development.

The COVID-19 pandemic prompted a rapid and significant shift to e-learning across educational disciplines, with particularly profound impacts in fields that rely heavily on practical training such as nursing [29–31].

In our study, students reported a reduction in opportunities for practical training, affecting their confidence and competence. This aligns with literature that highlights the need for also simulated practice in e-learning nursing programs generally [32]. We consider this is worth mentioning even though simulation is not the focus of this paper. Furthermore, the abrupt nature of the shift to e-learning underscored the importance of technical support and accommodations, which have been a greater challenge in nursing education compared to other disciplines where theoretical content predominates. Our participants expressed frustration over insufficient support to handle technical issues, a concern also echoed in another study examining the transition to e-learning under pandemic conditions [13]. These specific challenges in nursing education necessitate targeted pedagogical adjustments to support both academic and practical learning in an unpredictable and digital learning environment.

This study’s findings contribute to the broader discourse on e-learning by exploring its specific implications within nursing education during an unexpected global health crisis [31, 33–36]. This study is on e-learning during the COVID-19 pandemic and not on the shift per se, however the unique context of a sudden shift due to a pandemic presents particular challenges and opportunities that our study has explored in depth.

### Pandemic-specific findings and General E-Learning challenges

While many of the challenges identified in our study are consistent with general e-learning issues, the sudden and forced nature of the transition during the COVID-19 pandemic brought unique pressures. For instance, the rapid shift left little time for institutions to prepare optimal e-learning environments or for students to adjust to new learning modalities, exacerbating stress and anxiety. These conditions are distinct from planned e-learning strategies where students choose to enroll in e-learning courses, suggesting that future strategies should consider the abruptness of transitions and provide additional support during such times [37–39].

### Sociocultural perspective in E-learning

This study has illuminated various aspects of the transition to e-learning for nursing students and newly graduated nurses during the COVID-19 pandemic, with a particular focus on how this transition has affected their learning processes and professional development. In light of the challenges and opportunities presented by e-learning, it is fruitful to apply a sociocultural perspective, as described by Vygotsky [17], to deepen our understanding of these phenomena.

Vygotsky’s [17] theory of the Zone of Proximal Development (ZPD) provides a useful lens for understanding how learners can be optimally supported in an e-learning environment. The ZPD defines the difference between what a learner can do alone and what he or she can achieve with guidance and support from a more knowledgeable other [20]. During the pandemic, physical classrooms were replaced by digital platforms, and the traditional interaction between student and teacher was transformed. This necessitates that learning platforms and pedagogical methods be adapted to maximize educational support in the virtual learning environment, in line with Vygotsky’s principles [17].

A key takeaway from our study is the importance of scaffolding in e-learning environments. The concept of scaffolding, as discussed by Bruner [40], involves providing temporary support to students that is gradually removed as they become more independent. In the context of e-learning, this means creating structured, accessible, and predictable e-learning environments that can guide students through their educational journey. Our findings suggest that clear, consistent, and engaging instructional design is crucial for facilitating deep learning, where students engage critically and reflectively with the course material.

### The benefits of small group learning

Sociocultural theories also emphasize the importance of collaboration and dialogue in learning processes [17]. Although e-learning can be experienced as isolating, findings from our study indicate smaller group dynamics as a critical factor in enhancing e-learning effectiveness. This aligns with research by Wong (2018), who found that small groups facilitate more personalized interactions and deeper engagement, which is vital in a practice-oriented field like nursing. These groups allow for a transition from superficial to deep learning approaches, as defined by Marton and Säljö [18], by fostering critical engagement with material and collaborative learning experiences.

### Addressing technological and psychological challenges

Our study also brings to light the technological challenges that can impede e-learning [41]. As Kumar Basak et al. noted, effective e-learning platforms must be robust, user-friendly and aligned with educational goals [42]. The frequent technical disruptions experienced during the pandemic highlighted the necessity for reliable digital infrastructure and adequate support for both students and educators [43–45]. This is especially crucial in nursing education, where the stakes of training are inherently high due to the direct implications for patient care.

Additionally, the psychological and social challenges identified in our research reflect findings by Bdair, who highlighted the potential drawbacks of e-learning, such as inadequate interactions and increased feelings of isolation [8]. These challenges are particularly significant in nursing, where learning is not only about acquiring knowledge but also about developing empathetic patient care skills, which are best nurtured through direct human interactions.

### Integration of theory and practice

The challenges of integrating theoretical knowledge and practical skills in e-learning contexts, especially in nursing education, which is traditionally very practice-oriented, require innovative approaches to simulate practical experiences. This underscores the importance of ‘scaffolding’, where educators provide temporary support to students that they gradually withdraw as the students become more independent (Bruner [40]. E-learning platforms must be designed to support this pedagogical approach, clearly aligning with Vygotsky’s theories of learning through social interaction and supported exploration.

Integration with broader literature.

In comparing the challenges faced by nursing education during the rapid shift to e-learning with those in other disciplines, it becomes evident that the nature of nursing importantly amplifies these challenges [46]. Unlike disciplines primarily focused on theoretical knowledge, nursing education relies heavily on hands-on skills that are crucial for professional competence and patient care. The practical skills required in nursing, such as administering medications, performing physical assessments, and managing emergency situations, demand a level of tactile and sensory feedback that is inherently difficult, if not impossible, to replicate through e-learning platforms [2].

### Strengths

One of the significant strengths of this study lies in its timeliness and relevance. Conducted during an unprecedented global health crisis, it captures the immediate experiences and reactions of participants as they navigated the sudden transition to e-learning. This firsthand perspective is invaluable, offering real-time insights into the resilience, innovation, and adaptability of students and educators under crisis conditions.

Furthermore, this study systematically explores a wide range of themes related to e-learning in nursing education, addressing both the challenges and opportunities presented by this modality. By focusing on specific themes such as technological reliability, psychological impact, and pedagogical effectiveness, the research provides a detailed and balanced view of how e-learning can be optimized in nursing education. The use of qualitative methods enriches the data, allowing for a depth of understanding that can inform future educational strategies and interventions.

### Limitations and future research directions

Despite these strengths, the study has limitations that must be acknowledged. First, the sample is not representative of all nursing students globally or even across Europe. The participants were selected from specific geographic and educational settings within Norway, which may limit the transferability of the findings to other regions or educational contexts. External validity should be handled cautiously. In applying a holistic view, we have taken into account connections and influencing environments [47].

Another potential limitation is related to the rapidly evolving digital landscape. The digital landscape in general and e-learning platforms in particularly evolve rapidly. Therefore, the challenges faced during the initial phase of the pandemic might have differed from those faced later as institutions, students, and educators became more accustomed to digital teaching methods.

These limitations suggest that while this study has provided valuable initial insights into the challenges and possibilities of e-learning in nursing education during a crisis, further research is needed to understand the implications and to develop more-robust e-learning strategies for nursing education.

To build upon this study and address the identified limitations, the following research directions are proposed:

Long-term Perspective: There is a need for longitudinal studies that follow the development of e-learning in nursing education over time. This will help understand the long-term implications of digital teaching methods and how they can be improved for future crisis situations.

Technological Development: Research should focus on how the rapidly changing digital landscape affects the e-learning experience. This includes examining new technologies and platforms that can enhance the efficiency and user-friendliness of e-learning in nursing education.

Pedagogical Strategies: It is important to develop and test robust pedagogical strategies that effectively integrate e-learning. Future studies should explore various teaching methods and their impact on learning outcomes for nursing students.

Interactive and Immersive Technologies: Investigate the use of interactive and immersive technologies such as virtual reality (VR) and simulations in nursing education. Studies should assess how these technologies can complement traditional teaching and improve practical skills.

By exploring these research directions, future studies can contribute to enhancing the effectiveness and relevance of e-learning in nursing education and ensure better preparedness for future crisis situations.

### Implications for nursing education

Based on our research, educational institutions should consider the following strategies to enhance e-learning in nursing education:

*Implement Robust Scaffolding*: Develop and maintain structured, engaging, and accessible e-learning environments that provide the necessary support for students to achieve deep learning. This includes clear guidelines, consistent course materials, and active learning opportunities that guide students towards independence.

*Utilize Small Groups*: Promote the use of small groups in e-learning courses to enhance interaction and engagement. This approach not only supports deeper learning but also helps in developing the critical communication and teamwork skills essential for nursing.

*Invest in Technology and Support*: Ensure that the technological infrastructure supports seamless e-learning experiences. This includes reliable internet access, intuitive learning management systems, and prompt technical support to address issues as they arise.

*Continuous Professional Development for Educators*: Equip educators with the skills and tools necessary to effectively facilitate e-learning. This includes training in digital tools, pedagogical strategies for digital teaching, and methods to engage and assess students remotely.

*Monitor and Adapt Strategies*: Regularly review and adapt e-learning strategies based on feedback from students and educators, ensuring that the educational offerings meet the evolving needs of the nursing profession.

## Conclusion

The rapid shift to e-learning presented by the COVID-19 pandemic has posed challenges, such as technological, psychological and social aspects, it also offers an opportunity to rethink and enhance how nursing education is delivered. By understanding and implementing effective pedagogical e-learning strategies such as scaffolding and small group learning, educational institutions can better prepare nursing students for their crucial roles in healthcare. This study contributes to the body of knowledge on digital education and serves as a foundation for future research aimed at optimizing e-learning in nursing education.

## Electronic supplementary material

Below is the link to the electronic supplementary material.

Supplementary Material 1

Supplementary Material 2

## Data Availability

Availability of data and materialsTo access the dataset used and analysed during the current study, please contact the corresponding author.
