# Supplementary material for: Digital learning in nursing education: lessons from the COVID-19 lockdown
Source: BMC Nurs. 2024 Sep 11;23:646. doi: 10.1186/s12912-024-02312-1 (PMC11391838; doi:10.1186/s12912-024-02312-1)
Supplement: Supplementary file 1 — Supplementary Material 1 [file 12912_2024_2312_MOESM1_ESM.docx]

Interview with Students

The conversation aims to delve into four main areas:

I. Experiences with Clinical Practice Periods Consisting of Physical Presence and Digital Reflection Sessions During the COVID-19 Pandemic:

- Initial Discussion: Share your experiences with physical presence in clinical practice periods during the pandemic:
  - Your perceptions of being physically present in clinical settings during a pandemic.
  - How the learning outcomes of various clinical practice periods were achieved.
  - Guidance and support received from contact nurses/other nursing staff.
  - Interaction and follow-up by teacher advisors during practice periods when they couldn't be physically present.
  - If there were absences during practice periods, were alternative arrangements provided to compensate for the absence? (COVID-related tasks, absence assignments).

II. Experiences with Digital Gatherings During Clinical Practice Periods (e.g., Reflection Sessions):

- Preparation for these sessions.
- Your sense of responsibility in the preparation.
- Reflection, discussions with peers and teacher advisors on Zoom.
- Methods used (Reflection sessions in breakout rooms, submission of assignments).
- Experiences using Canvas as a learning platform in clinical studies.
- Any technical challenges encountered and their nature.
- Technical support for potential technical issues.
- Engagement, involvement, and enthusiasm in reflection group sessions on Zoom.
- Specific factors that either facilitated or hindered participation and learning.
- Perception of professional growth over time.

III. Reflections on End Competence Resulting from Clinical Studies in a Pandemic:

- General thoughts on the final competence due to clinical studies being conducted in a pandemic.
- Preparedness for soon functioning as officially recognized nurses.
- In the digital approach, can some aspects replace clinical practice? Thoughts on what can and cannot be substituted and the reasons.

IV. Simulation in Education:

- Experiences with the use of simulation in education:
  - Before clinical practice periods.
  - During clinical practice periods.
  - Learning processes involving simulation.
- How experiences in simulation are transferred to clinical practice and vice versa:
  - The impact of simulation activities before a clinical practice period on learning during clinical practice.
  - The influence of simulation activities during a clinical practice period on subsequent learning.
  - The effect of ongoing clinical practice on learning in simulations conducted during the clinical practice period.
- Reflections on Using Simulation as a Substitute for Parts of Clinical Studies:
  - Thoughts on substituting parts of clinical studies with simulation, regardless of the pandemic.
    - Methods and scenarios:
      - Practical skills.
      - First aid and CPR.
      - Medication management.
      - Basic needs scenarios in the first year.
      - Remote digital follow-up.
      - Acute situations in specialist healthcare in the third year.
      - Body Interact (Clinical Observations-GSKUV).
      - Acute situations in home care services in the fourth year.
    - Experiences from various clinical periods that suggest simulation could provide better learning outcomes than actual practice.
    - Overall experiences with the use of simulation.
