# Supplementary material for: Digital learning in nursing education: lessons from the COVID-19 lockdown
Source: BMC Nurs. 2024 Sep 11;23:646. doi: 10.1186/s12912-024-02312-1 (PMC11391838; doi:10.1186/s12912-024-02312-1)
Supplement: Supplementary file 2 — Supplementary Material 2 [file 12912_2024_2312_MOESM2_ESM.docx]

Interview with Graduated Nurses: Nurses who graduated in 2021

The aim of this project is to understand your perspectives and experiences with the teaching approach in alternative clinical practice from March 2020 to 2021, due to restrictions during the outbreak of the COVID-19 pandemic.

The conversation will focus on three main areas: I. Experiences with clinical practice periods consisting of a combination of (or substitution for) alternative practice (digital solutions) and physical presence during the COVID-19 pandemic. II. Reflections on your end-competence as a result of clinical studies during a pandemic. III. Reflections on the use of simulation, and whether the standard curriculum in simulation training can substitute for parts of clinical studies.

To start, please share your experiences with a combination of physical presence and the use of digital solutions in clinical practice periods: Workplace: .......................................... Age: ................... Cohort: .....................

- How were your clinical practice periods organized during the time you had practice during the pandemic?
- How did you experience being a student without the possibility of physical presence in the clinical practice period?
- How was the contact/follow-up with the teacher advisor during the clinical practice period when you couldn’t be physically present in practice?
- Experiences of stress while being in practice during a pandemic (what, how, consequences for learning) e.g., in relation to infection control.
